# A Comparative Review of Six Invasive *Nassella* Species in Australia with Implications for Their Management

**DOI:** 10.3390/plants10061036

**Published:** 2021-05-21

**Authors:** Talia Humphries, Singarayer K. Florentine

**Affiliations:** Future Regions Research Centre, School of Science, Psychology and Sport, Federation University Australia, Mount Helen, VIC 3350, Australia; taliajae@hotmail.com

**Keywords:** *Nassella*, weeds, management, invasive species

## Abstract

Two *Nassella* species, *Nassella trichotoma* and *Nassella neesiana*, have significantly reduced the carrying capacity of Australia’s south-east rangelands and agricultural systems. It is, therefore, of considerable concern that four other *Nassella* species have also become naturalised in Australia, and are noted to share many of the ecological features of the two currently widespread species. This paper reviews the distribution, ecology, and impacts of all six *Nassella* species, which are currently naturalised in Australia, and makes recommendations toward a blanket *Nassella* control program. The review highlights observed similarities between the species, including the time of flowering, seed type, germination requirements, and growth morphology. These common factors support the possibility that an integrated *Nassella* control program could be designed to integrate good grazing management with cultural control methods, such as soil cultivation, fire, and native plant competition, with treatments being implemented prior to the common annual seed maturation period. Notwithstanding the success of these integrated programs, it is recognised that seeds of all species may remain viable in the seedbank for up to 12 years, meaning ongoing monitoring and management will be required. To develop even finer control programs, further research into the ecology of these *Nassella* species is recommended to determine any additional weak spots in these species’ defences, and to subsequently develop and apply novel integrated control methods that target all six species.

## 1. Introduction

The displacement of productive pastures by unpalatable and inedible invasive species poses one of the biggest threats to the efficiency of Australian economic rangeland systems. Weed species cost Australia approximately $3.9 billion annually for control actions and in lost production; therefore, research into the ecology of these widespread weeds, as well as those recognised as being still in their establishment phase, is critical for protecting the nation’s economic and environmental assets. In Australia, two species belonging to the genus *Nassella*, *Nassella trichotoma* (Nees) Hack. ex Arechav. and *Nassella neesiana* (Trin. & Rupr.) Barkworth, are considered currently to be the most damaging species for reducing carrying capacity in Australian grazing systems due to their competitiveness, unpalatability, high fecundity, and the severe injuries they can cause livestock. These two species annually cost Australia’s grazing industry tens of millions of dollars in continuous control efforts and lost production. It has been estimated that *N. trichotoma* alone costs the New South Wales wool industry $40.1 million annually [1].

In addition to the two recognised *Nassella* species, there are four other *Nassella* species that are now naturalised in Australia; these are *Nassella hyalina* (Nees) Barkworth, *Nassella tenuissima* (Trin.) Barkworth, *Nassella charruana* (Arechav.) Barkworth, and *Nassella leucotricha* (Trin. & Rupr.) R. W. Pohl, and while they have not to date become widespread, they possess many biological similarities to *N. trichotoma* and *N. neesiana*, making their presence a significant concern [2]. Globally, there are over 100 species within the genus *Nassella*, which share the common feature of being long-lived (perennial) tussock- forming grasses that utilize the C_3_ photosynthetic pathway. This genus is native to North and South America [3], where disturbance events such as drought, fire, and grazing occur frequently; therefore, *Nassella* species are well adapted to these environmental pressures.

Due to their robust biological attributes, once they are established, these *Nassella* species are difficult to control, and in many cases introduced disturbance caused by management attempts such as burning or grubbing paradoxically promote their dominance in an area [4]. In the face of this significant challenge to control these species, we suggest that a review highlighting the common ecological traits of these six species will be beneficial for developing more tightly focused management strategies. This review will, therefore, identify (i) the ecological traits that are unique and similar to the six *Nassella* species in Australia and describe how these features may contribute to their establishment, spread, and resistance to control efforts; (ii) whether the ecological similarities between these six *Nassella* species can allow for the development of an overarching *Nassella* control program; and (iii) which areas require further research to contribute to the confident development of such an integrated control strategy.

### Species Overview

This section will detail the known and potential distribution, ecology, and ecological impacts for each of the six naturalised *Nassella* species in Australia. Table 1 compares the information relating to the current and potential distribution of each species, while Table 2 compares their ecological features.

## 2. *Nassella trichotoma* (Serrated Tussock)

### 2.1. Distribution

*Nassella trichotoma* was inadvertently introduced to Australia in the early 1900s but was not identified until 1938, where it was found growing in Yass in New South Wales [5]. It is suspected that this weed was introduced through contaminated fodder transported from New Zealand, and once authorities were alerted to its presence, it was soon also identified in Victoria in 1954 and Tasmania in 1965 [6,7]. To date, it has invaded an estimated 130,000 hectares throughout New South Wales, the Australian Capital Territory, Victoria, and Tasmania, however recent climatic modelling suggests it has a potential distribution of 32 million hectares throughout south-east Australia [5,8,9]. This *Nassella* species is known to be able to occupy a wide variety of environments, including native pastures and grasslands, coastal areas, open woodlands, and roadsides.

### 2.2. Ecology

The unpalatable, sclerophyllous leaves of *N. trichotoma* are fine and tightly rolled, and have a serrated texture that is felt when running the fingers from the tip to the base of the leaves [10]. *Nassella trichotoma* offers poor nutritional value to livestock as it has extremely low crude protein content of 3–12% [11] and between 74–86% fibre content [12,13]. Livestock that have been forced to graze on this species quickly lose condition, and in some cases leaves of this plant have been found in the stomach of starved sheep [11]. Because their leaves are sclerophyllous, this adaptive trait allows plants to better resist drought, grazing, or low soil nutrient levels, particularly in environments that are low in phosphorus [13]. Sclerophyllous leaves have reduced palatability, protecting them from grazing herbivores [14]. It is noted that the production of sclerophyll requires an energy trade-off that makes the re-growth of their leaves slower than those of non-sclerophyllous leaves [13], and as a consequence palatable grasses are frequently observed to be more competitive and faster growing than contending unpalatable grasses under favourable conditions. However, Distel et al. [13] found this not to be the case for *N. trichotoma*, where it was observed to produce the same or greater biomass than the palatable pasture grass *Stipa clarazii*. This suggests that *N. trichotoma* is likely to also be competitive with other productive grasses in the context of developing a grazing pasture.

*Nassella trichotoma* exhibits a high tolerance to drought, which is attributed to its fine and fibrous root system, which is found predominantly within the top 20–30 cm of the soil profile [15]. This species invests energy into its root system early in its development, thus allowing it to withstand drought conditions even in its early stages [16]. Further, this rooting structure makes it difficult to remove by hand, making cultural control methods such as grubbing difficult [16].

*Nassella trichotoma* produces panicle inflorescences that have a slight purple tinge, and as with other *Nassella* species, produce seeds from both allogamy (open) and cleistogamous (closed) flower heads, with the latter being more prominent [17,18]. *Nassella trichotoma* produces up to 90% cleistogamous flowers, allowing small populations to grow quickly, as fertilization is not reliant on pollen transfer [19,20,21]. Cross-pollination occurs via wind for the open flowers, and it has been found that no pollinator symbiosis is required for these flowers [16,20]. Cross-pollination has been observed to spread the emerging resistance to the widely-used herbicide flupropanate in Victoria, with resistant plants being observed over three kilometres from the original resistant population [6,17]. In the southern hemisphere, *N. trichotoma* has the longest flowering period of the genus, with inflorescences being observed from August through to March [22]. It is estimated that an individual plant of this species can produce up to 140,000 seeds per year [16,23].

*Nassella trichotoma* seeds are rounded and can be yellow to purple in colour. They have a mostly straight, off-centre awn reaching up to 3 cm in length, which assists the seed’s defence by burying it into the soil, protecting it from environmental stress and early devitalization [8,24]. These seeds also have small hairs on the callus to assist them in attaching to animals for zoochory dispersal [5,8]. However, the primary and most problematic dispersal mechanism for *N. trichotoma* is anemochory and hydrochory [16]. The seeds remain in the panicle, which detaches with ease from the parent plant on windy summer days and becomes airborne, allowing the seeds to travel up to 20 km from the parent plant. It has been estimated that seeds travel an average of approximately 8 km per day [16,24]. Seeds of this species are also effective at travelling in rainwater and rivers, which led to it being known colloquially as Yass River Tussock when first identified in Australia, since it was dominant along the waterways in the Yass region [8,25]. The seeds rarely germinate immediately, but rather have a brief dormancy period, with germination occurring in bursts during autumn and spring [26] or following heavy rainfall events [6]. *Nassella trichotoma* seeds have been found to occupy the top 2.5 cm of the soil profile, with minimal (1%) seeds being detected below this depth [26,27]. Seeds that are buried below 2.5 cm are not exposed to the environmental signals that stimulate germination, and these seeds can remain viable in a permanent dormant state for several years [26]. However, the majority of the seeds buried within the top 2.5 cm of the soil profile will germinate within their first year as long as water is available and where germination can be stimulated by light, alternating temperatures, and high heat. Somewhat surprisingly, germination can also occur in complete darkness [28], and germination for *N. trichotoma* is not impeded by moderate salinity levels or by varying soil pH levels, suggesting that poor quality soils and less than ideal germination environments can still support establishment of these weeds. 

### 2.3. Impact

This weed has severely low nutritional value and the ability to outcompete more palatable species in grazing environments; these features make this *N. trichotoma* one of the worst threats to carrying capacity in Australian rangelands. Unpalatable grasses divert nutrients away from the leaves to other growth processes, allowing them to be more effective grazing avoiders [13]. As indicated earlier, *Nassella trichotoma* is known to invest in its root systems early in life, demonstrating that it is well adapted to maximise water uptake during rainfall events and able to outcompete other species when water is limited [29]. These roots make removal through grubbing time consuming and labour intensive, which is why herbicides are the favoured control method for this species. Flupropanate is a semi-selective residual herbicide that kills *N. trichotoma* seedlings as they emerge, although its over-reliance has resulted in resistant populations in Victoria [30,31].

This weed produces large numbers of seeds, which can travel considerable distances by wind and water dispersal, and aided by its ability to attach to machinery or clothing, can be carried to unanticipated areas. Mass germination events are often directly associated with disturbances that can reduce competition, such as fire events. The dense formation of the leaves also protects its base from heat, allowing it to quickly re-sprout. Further, this weed has high sclerophyll content, which alters natural fire behaviour, resulting in more intense burns that have been observed to kill otherwise fire-tolerant native species [32]. Fire events are likely to become more frequent and intense under projected climate change, which could further promote *N. trichotoma’s* spread. For all these reasons, *N. trichotoma* is a prohibited species, being listed in Australia as a Weed of National Significance (WoNS). It is categorised, along with other WoNS, as a significant threat to socioeconomic and environmental values because of its competitiveness, ability to spread, and its impacts on human and livestock wellbeing.

## 3. *Nassella neesiana* (Chilean Needlegrass)

### 3.1. Distribution

*Nassella neesiana* is native to the South American countries of Argentina, Bolivia, Chile, Ecuador, Uruguay, and Brazil. It was first identified in Australia in 1934 in Victoria, followed by New South Wales in 1944. It is also naturalised in South Australia, the Australian Capital Territory, Queensland, and Tasmania [33]. It is a temperate grass species limited to environments with 500 mm annual precipitation. As a temperate grass species, it is limited to environments with 500 mm annual precipitation, and while soil type is not a limiting factor, it does not grow well in waterlogged areas [34]. Whilst this species is the widest spread of all the *Nassella* species in Australia, currently occupying over 40 million hectares [35,36], climatic modelling suggests that up to 180 million hectares will be climatically suitable for its establishment [37].

### 3.2. Ecology

Unlike *N. trichotoma*, the leaves of *N. neesiana* are flattened with defined ribs and short hairs, which give it a course, rough texture [10]; it is considered a suitable pasture species for grazing [38]. An analysis of the nutritional value of this species revealed that it had between 12.7 to 16.6% crude protein levels and 58 to 66% digestible dry matter, making it only slightly less palatable than *Festuca elatior*, which is considered to be a favourable pasture species [38]. It has been observed to be grazing-tolerant, with grazed leaves regrowing rapidly, assisted by increasing production of new shoots [10]. It has a fibrous and shallow root system and is an aggressive competitor for water, even in its juvenile form [36]. 

Inflorescences emerge between November and March in Australia, and during this time, *N. neesiana* has a distinct purple colouration. It produces both chasmogamic and cleistogamic flowers, and open flowers are wind-pollinated [18]. *Nassella neesiana* seeds are cylindrical and have a sharp, hooked tip. They are distinguishable by the presence of a corona where the awn and seed meet. *Nassella neesiana* panicles do not readily detach from the parent plant, and it was found that wind dispersed seeds often fall within one metre of the parent plant [35,39]. *Nassella* seeds have small, backwards-facing hairs on the callus that assist them in attaching to hair, wool, or fur of animals for zoochory dispersal [35]. *Nassella neesiana* seeds also have hairs on their awn that further enhance attachment ability, allowing them to stay attached to wool for up to five months, which could allow the seeds to be transported long distances with the movement of sheep and with shorn fleece [35]. A significant proportion of panicle seeds were shown to be devitalised by livestock ingestion in a previous study, which suggests this form of dispersal will be limited [35]. Additionally, the seeds can attach firmly to clothing, shoes, and machinery, which facilitates further dispersal [35]. 

*Nassella neesiana* also produces cleistogenes (stem seeds), which is advantageous in grazing situations and in frequently disturbed environments where the panicle seeds could be damaged prior to maturation [15,38,40]. Stem seeds lack awns, are always self-fertilised, and mature within the stem of the tussock rather than on panicles. Cleistogenes contribute to about a quarter of *N. neesiana’s* total seed density, which can be as high 22,200 seeds/m^2^ [38]. By comparison with panicle seeds, stem seeds were found to survive the digestive tract of Angus steers and subsequently germinate in higher proportions [35].

This species establishes large seedbanks, which can persist for many years. In Australia, seedbank densities have been recorded as high as 11,377 seeds/m^2^ [40], and even higher densities have been observed in New Zealand [26]. It was suggested that a seedbank with a density of 7000 seeds/m^2^ would take 12 years to reduce to 10 seeds/m^2^ without additional seed input [40]. This suggests that in areas where eradication has been seemingly achieved, the seedbank would require monitoring for over 12 years to prevent re-establishment of this species. As with *N. trichotoma*, the majority (99%) of the seeds remain within the top 2.5 cm of the soil profile [26], and it is likely that fluctuations in temperature and soil moisture promote germination [40]. Primary dormancy has also been observed for this species, with low germination occurring within the first three months of freshly harvested seeds, while two-year-old seeds readily germinate [40].

### 3.3. Impact

While *N. neesiana* leaves are of suitable quality for grazing, their seeds are sharp and can cause severe injury to livestock; thus, contamination with these seeds devalues the price of hide, wool, and meat [41]. The hooked shape of the seed, coupled with its hairy surface, promotes their attachment to passing animals for dispersal. The awn further assists attachment by burrowing the seed to into the skin or eyes of the animal, resulting in the formation of skin calluses and blindness [33,42]. This means that grazing areas with high densities of this weed become unusable during spring and summer months when the plant is in seed [43]. *Nassella neesiana* plants often grow close together, with their tussocks forming leaves that quickly shade the soil and repress the establishment of other species, allowing it to quickly become a monoculture [44]. Significant reductions in invertebrate species richness has also been observed in environments invaded by this weed [44].

Despite *N. neesiana* not effectively being dispersed by wind, it is more widespread than *N. trichotoma*. This is most likely due to human-assisted dispersal abetted by the movement of contaminated livestock or fodder. This species is well adapted to frequent environmental disturbances, including grazing, drought, and fire, often becoming dominant after these events [35,42]. McLaren et al. [45] found that on average, *N. neesiana* costs up to $118.75 per hectare to control on grazing lands, and the subsequent loss in selling capacity during the spring and summer months would clearly result in further losses. As this species could occupy up to 180 million hectares and has already caused significant damages to the economy, the environment, and animal welfare, *N. neesiana* is on Australia’s WoNS list.

## 4. *Nassella tenuissima* (Mexican Feathergrass)

### 4.1. Distribution

*Nassella tenuissima* is native to Texas, New Mexico, Argentina, and Chile. It was first identified in Australia in 1996, where it was misidentified and sold as Elegant spear grass (*Austrostipa elegantissima* (Labill.)) in a Sydney nursey [46]. Two years later, it was also detected as being sold in a nursey in Victoria’s Mount Macedon, which led to an investigation that found two other Victorian nurseries carrying this species [15,46]. An estimated 4000 *N. tenuissima* plants were sold, resulting in over 400 infested locations in Victoria alone [47]. Efforts were made to recall the sold plants, with approximately half being returned and destroyed [47]. While this species occupies only a very small area to date, climatic modelling has suggested that 100 million hectares are suitable for its establishment, including central Queensland and the arid zones of the Northern Territory and Western Australia [36]. Because the authorities acted quickly to trace and destroy the plants sold, *N. tenuissima* has not yet become widespread in Australia, with only small numbers of plants being recorded in New South Wales, Queensland, Western Australia, Tasmania, and Victoria. The majority of plants are thought to be located in the surrounding suburbs of Melbourne [48]. Due to its invasive properties, *N. tenuissima* is a prohibited weed in all states and territories in Australia.

### 4.2. Ecology

*Nassella tenuissma* has fine, tightly rolled, sclerophyllous leaves that can reach up to 70 cm in length. The leaves are very similar in appearance to *N. trichotoma*, but stand more upright and lack the serrations felt on *N. trichotoma* leaves. It has been reported that *N. tenuissima* leaves are less palatable than *N. trichotoma* within their native range [49]. An analysis of *N. tenuissma* green leaves revealed low levels of nitrogen (1.2%), high levels of lignin (8.1%), as well as high C/N ratios [49]. The root system is also very similar to other *Nassella* species, being shallow and fibrous, allowing for effective water uptake in environments with sporadic rainfall. *Nassella tenuissima* has the capacity to re-sprout if their wiry roots are not fully removed or treated [18,50], making mechanical removal problematic. 

Their inflorescences are slender and compact panicles measuring 8–50 cm in length. Panicle production occurs from September to December [51]. The panicles usually do not exceed the height of their leaves and the same panicle can produce both chasmogamic and cleistogamic flowers. For chasmogamic flowers, pollination is achieved through wind alone, while cleistogamic flowers self-fertilise, and these often mature earlier in the season [15,52]. While the panicles do not readily detach from parent *N. tenuissima*, their seeds are the smallest of all the *Nassella* species, which allows them to be effectively dispersed by wind [53]. *Nassella tenuissima* seeds are similar to *N. trichotoma* in colour and awn shape, however their lemma is more cylindrical and the awn is often longer. A single mature *N. tenuissima* plant can produce between 70,000–100,000 seeds annually, with up to 90% of the seeds germinating within their first two years, however small numbers can persist in excess of seven years [47]. In addition to the high seed production, vegetative regeneration has also been reported for this species with the presence of a bud bank. *Nassella tenuissima* produces 12 to 20 active buds per tiller or culm annually, allowing for vegetative regeneration after disturbance events [54].

A germination study conducted on the *N. tenuissima* seeds collected from the Mount Macedon nursery had only 20% germination after exposure to 23 °C and a photoperiod of 16 h light and 8 h dark, suggesting low viability of these seeds [46]. The low viability achieved in this study may have been a result of shallow physiological dormancy, as seen in other *Nassella* species [46]. To date, no research into seed dormancy for this species has been conducted, and it is plausible that this has contributed to this study’s low viability. When considering that this species has the capacity to produce up to 100,000 seeds per plant annually, even a low germination of 20% could have a significant impact. The seeds are light and could be transported great distances by wind [55]. Further, because this species relies not only on seeds for reproduction but also a bud bank, it makes this weed resilient to most disturbances and control methods [56]. 

### 4.3. Impact

*Nassella tenuissima* has been described as ‘unpalatable’ within its native range [49]; this attribute provides a competitive advantage when vying with palatable grass species, as grazing animals will selectively avoid it. Further, these unpalatable leaves cycle slowly back to the soil, which results in alterations of the soil chemistry, ultimately affecting the soil quality, which facilitates *Nassella* establishment [49]. *Nassella tenuissima* has not been observed to be as aggressive as other *Nassella* species, and is often outcompeted by more palatable species, usually making this species less problematic in areas that have good grazing management [57].

However, climatic modelling has predicted that up to 100 million hectares would be suitable for this weed’s establishment in Australia, suggesting that it could potentially cost up to $39 million AUD annually if it becomes widespread. For these reasons, it has been declared a noxious weed requiring control in all states and territories.

## 5. *Nassella leucotricha* (Texas Needle Grass)

### 5.1. Distribution

*Nassella leucotricha* currently has limited distribution in Australia, with the majority of the populations being confined to Melbourne’s northern and western suburbs [5]. This species was first introduced to Victoria in 1934, and despite most of Victoria providing a suitable climate, it has not become widely dispersed. It has also been recorded as naturalised in South Australia, with the majority of this population being contained within the Onkaparinga valley [58], while small populations have also been identified in New South Wales and Tasmania [59]. Bioclimatic modelling suggests that under current climatic conditions, *N. leucotricha* has the potential to invade an estimated 4.8 million hectares, including most of Victoria, the south-eastern regions of New South Wales, and the Australian Capitol Territory, as well as the southern regions of Western Australia and South Australia [5]. Unlike most other *Nassella* species, *N. leucotricha* is able to thrive in warmer, drier climates, due to its high water-use efficiency. This attribute was clearly important for its original development, being indigenous to Texas, Oklahoma, and central Mexico [60], all of which have warm, dry climates.

### 5.2. Ecology

*Nassella leucotricha* has erect, mostly flat leaves that are up to 30 cm in length [61]. Whilst research into the grazing value of this species is limited, it has been reported to provide important fodder during the winter months within its native range [62]. As with the other *Nassella* species, *N. leucotricha* has a shallow and fibrous root system that allows it to evidence efficient water uptake in drought-prone environments [59]. Flowering begins in early spring and the inflorescences take the form of a panicle, with these reaching up to 25 cm in length [62].

Within its native range, *N. leucotricha* produces seeds via cross-pollination (open flowers) and self-fertilization (closed flowers) [59,63]. The panicle seeds of *N. leucotricha* are up to 10 mm in length, and as their common name ‘Texas needle grass’ suggests, they have sharp, pointed tips that can cause serious harm to grazing animals and livestock [10]. The seeds also have short bristles at their base, which assists in their attachment to animals for dispersal, and the needle-like seeds can burrow into the flesh, mouth, or eyes of the animals to which they attach [64]. The ability to burrow is further assisted by a 60 mm, twice-bent awn [5,59]. It has been suggested that these seeds could remain in the wool of a sheep for many months, as reported for *N. neesiana* [35]. Notwithstanding this observation, the role animals play in seed dispersal for this species is not well documented. Additionally, whilst cleistogenes have not been reported as present in Australia [10], their common presence within their native range should be taken into consideration when developing future management practices [59].

The seeds have been observed to have primary dormancy, and fresh seeds express low rates of germination [60]. Van Auken [60] identified that removing the lemma and palea increased the germination of freshly harvested seeds from 1% to 4% after 15 days of incubation at 25 °C and alternating 12 h light and dark photoperiods. In the same germination treatment, seeds that were harvested nine years prior to the treatment exhibited 84% germination [60]. Germination has been observed to be improved by exposing the seeds to increased temperature regimes of 25 to 35 °C for three to six months [60]. These results compliment the germination ecology observed in its native range, whereby germination and growth begin at the end of autumn and continue into late spring [59].

### 5.3. Impact

Whilst there is currently no detailed information regarding the nutritional value of *N. leucotricha*, within its native range it is considered to be a valued pasture grass for winter grazing, as the summer-growing C_4_ grasses are often dormant at this time [10]. In Australia, regardless of its palatability, this species still poses a high threat to pasture systems [63], reducing their carrying capacity, particularly in summer, when it produces unpalatable inflorescences and sharp seeds that can cause serious injury to livestock [64]. The weed also outcompetes important summer pasture species, making areas dominated by this weed unfit for summer grazing, and consequently it was considered to be the seventh worst weed on the DEH Alert List [65].

Its current distribution in Australia is within the temperate zone [5], which is different from the warm and dry climate it occupies within its native range [60]. Despite this, *N. leucotricha* achieves maximum growth during the winter season within its native range, and climate modelling has suggested that it is suited to all Victorian areas, as well as southern South Australia, southern Western Australia, and south east New South Wales [5].

Disturbance events such as drought, grazing, and fire have been observed to promote the density of *N. leucotricha* [59,66]. It has high drought tolerance attributed to its fibrous root system, which allows it to take advantage of sporadic rainfall, and it exhibits reduced growth during summer. The seeds avoid germination under unfavourable conditions due to natural primary dormancy, which is broken by cooler temperatures [60]. The potential presence of cleistogenes could allow for quick recovery of the population after fire, and if ingested by livestock, they can be transported over long distances to invade new areas [59].

## 6. *Nassella charruana* (Lobed Needlegrass)

### 6.1. Distribution

*Nassella charruana* is native to the South American countries of Argentina, Brazil, Paraguay, and Uruguay. It was imported to Australia in 1945 as part of the Commonwealth Plant Introduction program as a potential pasture species [67]. Currently, *N. charruana* is only found in Victoria, where it occupies approximately 20 hectares. It has been observed to occur in open woodlands and degraded grasslands, and has been observed to displace other *Nassella* species [68]. Climatic modelling suggests this weed could occupy from 440,952 to 600,000 hectares in Australia, with most of Victoria being climatically suitable, along with temperate areas of New South Wales, South Australia, Australian Capital Territory, and Tasmania [5,69].

### 6.2. Ecology

The leaves of *N. charruana* are thin (0.5–1 mm wide) and stiff, giving this species a tall tufted appearance [68,70]. The xeromorphic leaves improve its tolerance to drought conditions, and it has been observed to regrow profusely after fire and grazing [71]. The root system is dense and extensive and has a similar appearance to other *Nassella* species [72]. *Nassella charruana* has been observed to form rhizomes, which could promote this species’ ability to access water [68]. A preference for shallow, fertile soils has been observed for this species, which could put pasture systems at greater risk of invasion than most natural sites [73].

The seeds appear as similar in shape to *N. neesiana*, and they are also sharp with a hooked tip, allowing effective attachment to passing animals [10,68]. The seeds cause injury to livestock by burrowing into their skin, mouth, and eyes [68]. Attachment to animals has been identified as the primary mode of dispersal (zoochory) [74], as the wind has not been observed to distribute their seeds.

Unlike *N. neesiana*, *N. charruana* has been observed growing in wet soil depressions and is most competitive in clay soils [5]. Within its native range and under grazing disturbance, this species was observed to increase in humid mesophytic meadows [73], suggesting that ample water availability is important for its germination. Light may also be an important germination cue, as plants are usually found growing in full sunlight or part-shaded areas [5]. To date, no seed ecology studies have been conducted for this species, and little is known about its seed dormancy, seed longevity, seedbank density, or seed output [74].

### 6.3. Impact

*Nassella charruana* shares the unpalatability traits of *N. trichotoma* and also the injurious seeds of *N. neesiana*, and within its native range, this species is considered highly competitive, even displacing other *Nassella* species [5,75]. For these reasons, *N. charruana* poses a significant threat to temperate rangelands, particularly those with high rainfall and soil fertility [69]. Currently, this weed is only found in Victoria, and the population has been contained [68]. Despite being introduced in the 1940s, it was not considered a potential weed species until 1995, but is now on the ‘Weed Alert List’ of the Commonwealth Department of Environment and Heritage and is recommended for containment and eradication [65]. It is also on the ‘Sleeper Weeds List’, as very little it still known of its ecology in Australia, and long lag times have been observed in *Nassella* species previously, as seen in the spread of *N. neesiana* in New Zealand, which was first identified in the 1940s [76]. This did not become a widespread problem until 1987. The difficulty in implementing eradication programs for this species is exacerbated by the lack of available knowledge on the seedbank and seed longevity of this species, making more detailed research essential for ongoing monitoring programs in treated areas.

## 7. *Nassella hyalina* (Cane Needlegrass)

### 7.1. Distribution

*Nassella hyalina* is native to Argentina, Uruguay, and Brazil. It was first identified growing in northern New South Wales in 1951, and later in central Victoria in 1964 [77]. The populations are contained within these areas, with the largest densities occurring in the western suburbs of Melbourne [5]. Climatic modelling suggests the potential distribution of this weed in Australia is up to 900,000 hectares in areas with high rainfall zones [5,78].

### 7.2. Ecology

The leaves of *N. hyalina* are mostly flattened to slightly in-rolled and reach lengths of up to 20 cm [66,79]. The leaves grow with a sparser habit than other *Nassella* species and the base of the plant has a clumped growth form that allows it to reach heights of up 120 cm [66,73]. It offers inferior forage quality compared to palatable pastures, however it has been seen to be grazed in the absence of more appropriate plants [10,75,79]. 

*Nassella hyalina* produces flowers and seeds in spring and summer, and reproduction has been observed to occur within a plant’s first year [79,80]. This species has been identified to produce both panicle and stem seeds [10,79]. It was described as being predominantly cleistogamous, which suggests most seeds are produced through self-fertilizing, closed flowers on the panicle, as well as those produced within the stem [75]. The number of seeds produced by a plant is unknown, nor is there available information on seed dormancy, germination cues, or longevity. The slender-shaped seeds are covered with short hairs and have a hooked tip that promotes their zoochory dispersal [10,79]. The panicle seeds have an awn that reaches up to 4.5 cm to assist their burial into the soil [79]. Cleistogenes are formed in the stem and play an important role in this species’ reproduction after disturbance events [74]. The stems containing the hidden seeds become loose after the panicle seeds have matured and are easily dislodged by animals, wind, or water, which allows for their dispersal [81].

### 7.3. Impact

While *N. hyalina* shares many similarities to *N. neesiana*, it is not widespread, despite 900,000 hectares being currently climatically suitable for its distribution [78]. If this species was to become widespread, it could pose a significant threat to rangelands and grazing systems, as this species offers poor quality fodder and the seeds can cause injuries to livestock. This would degrade the quality of meat, wool, and hide [79,81], resulting in significant reductions to carrying capacity, together with further losses related to actions taken to control the weed. Control is complicated by the presence of cleistogenes, which promote new growth following disturbances [5,79,81]. For these reasons, *N. hyalina* is on the ‘Weed Alert List’ of the Commonwealth Department of Environment and Heritage [65].

## 8. Management Implications

There are similarities between these six *Nassella* species with regards to their leaf grazing quality, reproduction timing, pollination, and germination cues [5,9,10,18,42,59]. While the palatability of these species varies, they are all considerably less palatable when compared to standard pasture grasses [10,38]. *Nassella trichotoma*, *N. charruana*, and *N. tenuissima* are of considerably low palatability, and if livestock are forced to graze these species, they quickly lose condition [82]. This avoidance by grazing animals allows for unpalatable grasses to establish in grazing situations, as they can grow undisturbed while palatable species are more frequently targeted by livestock [57]. However, under well-managed pasture systems, palatable species can be more productive and competitive, which reduces the growth of *Nassella* species [83], indicating that the maintenance of high native grass cover is an important preventative management technique [83,84]. 

Although drought periods occur frequently in the temperate regions of Australia, the hardened leaves and fibrous root structure allows these *Nassella* species to remain competitive under these otherwise adverse conditions [13,49]. The dense tussock formation of the leaves also protects the plant’s crown from frost and fire, and the literature suggests that fire promotes regrowth from the plant’s base and encourages seed germination [8,16,71]. When *N. trichotoma* seeds were exposed to radiant heat in a germination trial, significant increases in germination rate and percentage (100%) were observed [28,42]. Therefore, fire, as a single control method, would likely increase this weed’s dominance. Nevertheless, it could be used to significantly reduce above-ground biomass, but it would be then critical to establish competition immediately after burning. In this respect, resting a burnt site from grazing has been recommended for preventing establishment of dominance for unpalatable grasses [85].

Inflorescences appear in the spring and continue into the summer for all six *Nassella* species, which suggests that *Nassella* control programs should be implemented at the same time of year [43,79,80]. The available literature suggests that seed production is high, with pollination mostly occurring within cleistogamous flowers [17,18]. This demonstrates that if even a single plant establishes in a grazing system, it can add a significant number of seeds to the seedbank, as it does not require cross-pollination to produce large seed densities [40]. Seed bank densities of these *Nassella* species are often high, and it is suggested that the seeds can remain viable in the soil for many years, with reports of *N. tenuissima* seeds remaining viable for seven years [47] and *N. neesiana* seeds for 12 years [40]. This indicates that control programs need to monitor re-emergence from the seedbanks for at least 12 years, and that research into accelerating the devitalisation process of the seedbanks should be further investigated.

For *N. neesiana* and *N. trichotoma*, over 99% of their seedbanks are located within the top 2.5 cm of the soil profile [26]. As the other naturalised *Nassella* species have similarly shaped awns (Table 2), it could be speculated that the seeds of these species would also occupy the same portion of the soil profile. It has also been observed that emergence of *N. trichotoma* is significantly reduced at 4 cm burial depth [28], which may be similar for the other *Nassella* species. Therefore, in heavily invaded areas, deep soil cultivation to bury seeds could be useful, or alternatively topsoil removal could be an effective control option for removing the high density of *Nassella* seeds. Another method for reducing the seedbank would involve promoting a mass germination event to flush out the seedbank, then removing the germinated seeds through cultivation or herbicide. In this respect, primary dormancy has been observed for *N. trichotoma* [26,27], *N. neesiana* [40], and *N. leucotricha* [60], and since the other three *Nassella* species share similar climatic envelopes, it is likely that they too exhibit primary dormancy to prevent germination in the summer heat. The environmental cues that promote mass germination of these *Nassella* species, such as fire, could be implemented to promote seedling emergence, which can be then treated by follow-up management. 

Dispersal mechanisms differed between some of the species, with *N. trichotoma* and *N. tenuissima* predominantly using anemochory, while the other species rely heavily on zoochory (Table 2). *Nassella neesiana* seeds were found in the wool of sheep after five months of exposure [35], and since similar attachment features are seen on most of these *Nassella* species, long holding periods for any livestock in paddocks containing *Nassella* species would be recommended. Reducing the spread of the wind-dispersed species requires treating the plants and removing flowers prior to seed maturation, since if the seeds are not removed, wind has been observed to disperse seeds many kilometres from the parent plant [16]. While these natural dispersal methods are highly effective, human-aided dispersal is likely to also contribute to larger scale spread of these weeds [86].

Another difference between these *Nassella* species is the presence of stem seeds in *N. neesiana*, *N. leucotricha*, and *N. hyalina* [42,59,81]. These seeds add complications to control techniques that target the reduction of plant biomass, as methods such as slashing, mowing, grazing, or fire can promote their dispersal and germination. Good machinery hygiene is important for reducing the risk of spreading these species after implementing control techniques [87]. While stem seeds of *N. neesiana* were observed to survive the digestive tract of livestock, their viability was reduced and the seeds often passed within four days [40], suggesting holding periods for consumed cleistogenes would not need to exceed a week.

In Australia, there are no native *Nassella* species, which hampers efforts to find a suitable biological control [88,89]. To date, there have not yet been any successful introduction of a biological control agent, despite a variety of fungi, rusts, moulds, and nematodes having been investigated. The introduction of a biological control could greatly enhance the success of control programs for these species, since it is known that natural predators of *Nassella* are important factors for maintaining population densities within their native ranges.

## 9. Conclusions

*Nassella* species pose one of the biggest threats to Australia’s rangelands and temperate environments. They cause significant harm to livestock through the presence of sharp, needle-like seeds and unpalatable, indigestible leaves. These species are competitive in disturbed and degraded landscapes and aggressively invade areas that are overgrazed. They have high reproductive output, effective dispersal mechanisms, and persistent seedbanks, making their management an ongoing battle, even after they appear to have been eradicated. This review has highlighted the many similarities between the *Nassella* species that could allow for the development of an overarching *Nassella* control program for rangelands and grazing systems at risk within Australia. Further research into the ecology of these species should be considered, particularly those that are not yet widespread, as these species have many phenotypic similarities with *N. trichotoma* and *N. neesiana*, and thus could cause significant damage if they also become widespread.

## Figures and Tables

**Table 1:** Distribution and climate preferences of the six Nassella species naturalised in Australia.

	*Nassella trichotoma*	*Nassella neesiana*	*Nassella hyalina*	*Nassella leucotricha*	*Nassella charruana*	*Nassella tenuissima*
*Native Range*	Argentina, Chile, Brazil, Uruguay	Argentina, Bolivia, Chile, Ecuador, Brazil, Uruguay	Argentina, Brazil, Uruguay	Oklahoma, Texas, Mexico	Uruguay, Argentina, Brazil	Mexico, USA, Chile, Argentina
*First Identified in Australia*	1938	1934	1951	1934	1995	2008
*Australian Distribution*	130,000 ha currently invaded in NSW, VIC, ACT, and TAS.	432,157 ha have been invaded throughout ACT, NSW, SA, TAS, and VIC.	NSW and VIC.	Not widely distributed, largest population found in VIC, small numbers of plants in ACT, NSW and SA.	20 ha in Victoria.	Small numbers in VIC, NSW, QLD and WA.
*Potential Distribution*	32 million ha	41–180 million ha	0.9 million ha	4.8 million ha	0.6 million ha	14.1–100 million ha
*Preferred Vegetation Communities*	Dry coastal, lowland grassland, grassy woodland, dry sclerophyll forest, rocky outcrop	Lowland grasslands, grassy woodlands, riparian	Riparian, grasslands	Grasslands	Grasslands	Open woodlands, grasslands
*Legislation*	Weed of National Significance	Weed of National Significance	Alert List for Environmental Weeds, is declared a weed in SA	Declared a weed in SA and VIC	Alert List and Sleeper List for Environmental Weeds. It is declared a weed in VIC and ACT	Restricted invasive plant under the Biosecurity Act 2014

**Table 2:** The ecological characteristics of the six naturalised Nassella species in Australia.

*Ecological trait*	*Nassella trichotoma*	*Nassella neesiana*	*Nassella hyalina*	*Nassella leucotricha*	*Nassella charruana*	*Nassella tenuissima*
*Leaves*	Fine and tightly rolled, very unpalatable and sclerophyllous.	Flattened with defined ribs, offering adequate fodder quality.	Flattened leaves that offer low quality grazing.	Flattened leaves that can be grazed in winter.	Thin with a stiff appearance.	Fine and tightly rolled, very unpalatable and sclerophyllous.
*Roots*	Shallow, fine, and fibrous.	Fibrous and fast growing.	Fine and fibrous	Shallow, fine, and fibrous.	Fine, fibrous, and shallow, and rhizomes are present.	Fibrous and grow close to the soil surface.
*Inflorescence*	Purplish in colour and they emerge in spring. They are branched, 10–25 cm in length, and once seeds are mature, they detach readily from the plant.	Flowering begins in march, and these purplish panicles are between 10–25 cm in length. Panicle does not readily detach.	Panicles appear in spring, reaching up to 30 cm in length, and plants can seed in their first year.	Panicles appear in spring and produces flowers within first year.	Panicles appear in spring, do not readily detach from plant.	Panicles appear in spring, do not readily detach from plant.
*Seeds*	Small and rounded seeds with fine hairs present, the awn is off-centre.	Thin needle-like appearance, with a long awn and hooked tip. Awn and seed are covered with fine hairs. Also produces stem seeds.	Slender in shape with a hooked tip and short hairs. A 4.5 cm awn is present *n* panicle seeds. Stem seeds have been observed.	Produces both panicle and stem seeds. Panicle seeds have a hooked tip, short hairs and awn.	Needle-like shape with sharp hooked tip, hairs and awn present.	Small and light, awn and small hairs present.
*Primary Dispersal*	Panicle detaches in the wind, which carries the panicle containing the seeds great distances.	Attachment to passing animals/human or machinery. Stem seeds are dispersed by disturbances.	Attachment to passing animals/human or machinery. Stem seeds are dispersed by disturbances.	Attachment to passing animals/humans or machinery. Stem seeds are dispersed by disturbances.	Attachment to passing animals/human or machinery. Stem seeds are dispersed by disturbances.	The light seeds are dispersed most effectively by wind.
*Temperature*	These are restricted to temperate areas, as water is required for germination.	Restricted to temperate temperatures.	Restricted to temperate temperatures.	Restricted to temperate temperatures.	Suited to temperate areas and can tolerate waterlogged areas.	Best suited to temperate temperatures, but can tolerate warmer and drier climates.
*Habitat*	They can be found in most soil types, tolerant to moderate salinity, and soil pH is not a limiting factor	Soil type is not a limiting factor, although not found growing in waterlogged soils.	Best suited to fertile areas, it can tolerate waterlogged soils.	Prefers disturbed areas, and soil type is not a limiting factor.	Currently found in open woodlands and degraded grasslands.	Soil type is not a limiting factor, it prefers well drained soils with moderate fertility.
*Rainfall*	Requires 500 mm of annual rainfall or more.	Requires 500 mm of annual rainfall or more.	Requires 500 mm of annual rainfall or more.	Requires 500 mm of annual rainfall or more.	Requires 500 mm of annual rainfall or more.	Requires 300 mm of annual rainfall, very tolerant to environmental extremes.

## Data Availability

Not applicable.

## References

[1] Jones R.E., Vere D.T. (1998). The economics of serrated tussock in New South Wales. Plant Prot. Q..

[2] Martin T.G., Campbell S., Grounds S. (2006). Weeds of Australian rangelands. Rangel. J..

[3] Cialdella A.M., Sede S.M., Romaschenko K., Peterson P.M., Soreng R.J., Zuloaga F.O., Morrone O. (2014). Phylogeny of Nassella (Stipeae, Pooideae, Poaceae) Based on Analyses of Chloroplast and Nuclear Ribosomal DNA and Morphology. Syst. Bot..

[4] Lamoureaux S., Bourdôt G., Spafford H., Dodd J., Moore J.H. (2002). *Nassella trichotoma* (Nees) Arechav.: Seedling recruitment and survival in New Zealand. Weeds, Threats Now, and Forever? Proceedings of the Thirteenth Australian Weeds Conference, Victoria Park, WA, Australia, 8–13 September 2002.

[5] McLaren D.A., Stajsic V., Gardener M.R. (1998). The Distribution and impact of south/north American stipoid grasses (Poaceae: Stipeae). Plant Prot. Q..

[6] Campbell M.H., Vere D.T., Groves R.H., Shepherd R.H., Richardson R.G. (1995). *Nassella trichotoma* (Nees) Arech. The Biology of Australian Weeds.

[7] Parsons W.T., Cuthbertson E.G. (2001). Noxious Weeds of Australia.

[8] Atlas of Living Australia Weeds Australia Profiles—*Nassella trichotoma* (Nees) Hack. ex. https://profiles.ala.org.au/opus/weeds-australia/profile/Nassella%20trichotoma.

[9] Kriticos D., Lamoureaux S., Bourdôt G., Pettit W. (2004). Nassella tussock current and potential distributions in New Zealand. N. Z. Plant Prot..

[10] McLaren D.A., Stajsic V., Iaconis L. (2004). The distribution, impacts and identification of exotic stipoid grasses. Plant Prot. Q..

[11] Campbell M., Barkus B. (1961). The effect of fertilizers on the crude protein content and palatability of serrated tussock (*Nassella trichotoma* (Nees.) Hack.). Aust. J. Exp. Agric..

[12] Vere D.T., Campbell M.H. (1984). Economics of Controlling serrated tussock in the south-eastern Australian Rangelands. J. Range Manag..

[13] Distel R.A., Moretto A.S., Didoné N.G. (2007). Regrowth capacity in relation to defence strategy in *Stipa clarazii* and *Stipa trichotoma*, native to semiarid Argentina. Austral Ecol..

[14] Distel R., Didoné N., Moretto A. (2005). Variations in chemical composition associated with tissue aging in palatable and unpalatable grasses native to central Argentina. J. Arid Environ..

[15] Jacobs S., Everett J., Torres M. (1998). *Nassella tenuissima* (Gramineae) recorded from Australia, a potential new weed related to Serrated Tussock. Telopea.

[16] Osmond R., Verbeek M., McLaren D.A., Michelmore M., Wicks B., Grech C.J., Fullerton P. (2008). Serrated Tussock—National Best Practice Manual.

[17] Ramasamy S., Pritchard G., McLaren D.A., Bonilla J., Preston C., Lawrie A.C., van Klinken R.D. (2008). Field survey of flupropanate-resistant *Nassella trichotoma* in Victoria. Hot Topics in the Tropics, Proceedings of the 16th Australian Weeds Conference, Cairns Convention Centre, North QLD, Australia, 18–22 May 2008.

[18] Mapaura A., Canavan K., Richardson D.M., Clark V.R., Steenhuisen S.-L. (2020). The invasive grass genus *Nassella* in South Africa: A synthesis. S. Afr. J. Bot..

[19] Barret S.C.H., Charudattan R., Walker H.L. (1982). Genetic variations in weeds. Biological Control of Weeds with Plant Pathogens.

[20] Connor H.E., Edgar E., Bourdot G.W. (1993). Ecology and distribution of naturalised species of Stipa in New Zealand. N. Z. J. Agric. Res..

[21] Ramasamy S., McLaren D., Preston C., Lawrie A.C., Zydenbos S.M. (2010). Heritability of flupropanate resistance in *Nassella trichotoma*. New Frontiers in New Zealand, Together We Can Beat the Weeds, Proceedings of the Seventeenth Australasian Weeds Conference, Christchurch, New Zealand, 26–30 September 2010.

[22] CRC for Australian Weed Management, Commonwealth Department of the Environment and Heritage (2013). Serrated Tussock (Nassella trichotoma), Weed Management Guide.

[23] Invasive Species; Serrated Tussock; What Is Serrated Tussock? Tasmanian Government, Department of Primary Industries, Parks, Water and Environment. https://dpipwe.tas.gov.au/invasive-species/weeds/weeds-index/declared-weeds-index/serrated-tussock.

[24] CABI Datasheet: *Nassella trichotoma* (Serrated Tussock Grass). https://www.cabi.org/isc/datasheet/35726.

[25] Cross D.O. (1937). Yass river tussock. Agric. Gaz. N. S. W..

[26] Bourdôt G.W., Hurrell G.A. (1992). Aspects of the ecology of *Stipa neesiana* Trin. & Rupr. Seeds. N. Z. J. Agric. Res..

[27] Joubert D.C. (1984). The soil seed bank under *Nasella* tussock infestations at Boschberg. S. Afr. J. Plant Soil.

[28] Humphries T., Chauhan B.S., Florentine S.K. (2018). Environmental factors effecting the germination and seedling emergence of two populations of an aggressive agricultural weed; *Nassella trichotoma*. PLoS ONE.

[29] García A., Scarfó M.C., Loydi A., Distel R.A. (2020). Effect of competition intensity on recruitment of palatable and unpalatable grasses. Austral Ecol..

[30] McLaren D.A., Merton E., Pritchard G., Ramasamy S., Grech C.J., Bonilla J., van Klinken R.D. (2008). Evaluating the extent of serrated tussock (*Nassella trichotoma*) resistance to the herbicide, flupropanate in Australia. Hot Topics in the Tropics, Proceedings of the 16th Australian Weeds Conference, Cairns Convention Centre, North Queensland, Australia, 18–22 May 2008.

[31] Grech C., Mclaren D., Bennett H., Butler K. (2009). Effects of Flupropanate on Non-Target Species—Glasshouse.

[32] Sinclair S.J., Duncan D., Bruce M.J. (2014). Mortality of native grasses after a summer fire in natural temperate grassland suggests ecosystem instability. Ecol. Manag. Restor..

[33] VicFlora, *Nassella neesiana* (Trin. & Rupr.) Barkwork. https://vicflora.rbg.vic.gov.au/flora/taxon/24a0a8c1-38bb-45eb-bdb3-4dea32a0800f#:~:text=Chilean%20Needle%2Dgrass&text=Leaves%20glabrous%20or%20sparsely%20pubescent,often%20twisting%20together%20at%20maturity.

[34] Kabaš E., Ljubičić I. (2019). First record of *Nassella neesiana* (Trin. & Rupr.) Barkworth (Poaceae) in Croatia. BioInvasions Rec..

[35] Gardener M.R., Whalley R.D.B., Sindel B.M. (2003). Ecology of *Nassella neesiana*, Chilean needle grass, in pastures on the Northern Tablelands of New South Wales. I. Seed production and dispersal. Aust. J. Agric. Res..

[36] Grice T. (2018). Weeds of Significance to the Grazing Industries of Australia.

[37] Bourdot G.W., Lamoureaux S.L., Kriticos D.J., Watt M.S., Brown M., Zydenbos S.M. (2010). Current and potential distributions of *Nassella neesiana* (Chilean needle grass) in Australia and New Zealand. New Frontiers in New Zealand, Together We Can Beat the Weeds, Proceedings of the Seventeenth Australasian Weeds Conference, Christchurch, New Zealand, 26–30 September 2010.

[38] Gardener M.R., Sindel B.M., Whalley R.B.D., Bishop A.C., Boersma M.M., Barnes C.D. (1999). Chilean needle grass (*Nassella neesiana*)—What we know and where to next?. Weed Management into the 21st Century: Do We Know Where We’re Going? Proceedings of the 12th Australian Weeds Conference, Hobart, TAS, Australia, 12–16 September 1999.

[39] Faithful I., Hocking C., McLaren D., van Klinken R.D., Osten V.D., Panetta F.D., Scanlan J.C. (2008). An investigation of the effects of disturbance on the establishment of *Nassella neesiana* (Trin. & Rupr.) Barkworth (Poaceae) in an Australian native grassland. Proceedings of the Sixteenth Australian Weeds Conference.

[40] Gardener M.R., Whalley R.D.B., Sindel B.M. (2003). Ecology of *Nassella neesiana*, Chilean needle grass, in pastures on the Northern Tablelands of New South Wales. II. Seedbank dynamics, seed germination, and seedling recruitment. Aust. J. Agric. Res..

[41] McLaren D.A., Weiss J.E.R., Faithfull I., Grice T. (2018). Weeds of significance to grazing industries of Victoria. Weeds of Significance to the Grazing Industries of Australia.

[42] Atlas of Living Australia, Weeds Australia Profiles—*Nassella neesiana* (Trin. & Rupr.). https://profiles.ala.org.au/opus/weeds-australia/profile/Nassella%20neesiana.

[43] Agriculture Victoria Chilean Needle Grass. https://agriculture.vic.gov.au/biosecurity/weeds/priority-weeds/chilean-needle-grass.

[44] Faithful I., Hocking C., McLaren D., Zydenbos S.M. (2010). Chilean needle grass (*Nassella neesiana*) in the native grasslands of south-eastern Australia: Biodiversity effects, invasion drivers and impact mechanisms. New Frontiers in New Zealand, Together We Can Beat the Weeds, Proceedings of the Seventeenth Australasian Weeds Conference, Christchurch, New Zealand, 26–30 September 2010.

[45] McLaren D.A., Coventry R., Thorp J. (2002). Stipoid grasses as Weeds of National Significance. Plant Prot. Q..

[46] McLaren D.A., Whattam M., Blood K., Stajsic V., Hore R., Bishop A.C., Boersma M.M., Barnes C.D. (1999). Mexican feather grass (*Nassella tenuissima*) a potential disaster for Australia. Weed Management into the 21st Century: Do We Know Where We’re Going? Proceedings of the 12th Australian Weeds Conference, Hobart, TAS, Australia, 12–16 September 1999.

[47] Moran N.P., Rowles A.D., Constantine A.B., Johnson S., Weston L., Wu H.W., Auld B. (2018). 10 years after Victoria’s major Mexican feather grass incursion: Assessing eradication methods and germination behaviour. Weed Biosecurity—Protecting Our Future, Proceedings of the 21st Australasian Weeds Conference, Sydney, Australia, 9–13 September 2018.

[48] The Australasian Virtual Herbarium, Occurrence Records—Genus *Nassella*. https://avh.ala.org.au/occurrences/search?taxa=nassella#tab_mapView.

[49] Moretto A.S., Distel R.A. (2002). Soil nitrogen availability under grass of different palatability in a temperate semi-arid rangeland of central Argentina. Austral Ecol..

[50] PIER *Nassella* *tenuissima*. http://www.hear.org/pier/species/nassella_tenuissima.htm.

[51] VicFlora *Nassella tenuissima* (Trin.) Barkworth. Royal Botanic Gardens Victoria. https://vicflora.rbg.vic.gov.au/flora/taxon/cab4b64d-7a50-4a5a-a780-210010b647c9.

[52] Connor H.E., Ford K. (1996). *Stipa tenuissima*, and name changes in stipoid grasses. Weed Identif. News.

[53] Marshal A., Williams N., Marshall A., Morgan J. (2015). Land of Sweeping Plains: Managing and Restoring the Native Grasslands of South-Eastern Australia.

[54] Russell M.L., Rector B.S. (2007). Mexican Feathergrass.

[55] Conolly J., Taylor S., Johnson S., Weston L., Wu H.W., Auld B. (2016). Mexican feather grass (*Nassella tenuissima*) control—The ACT experience. Weed Biosecurity—Protecting Our Future, Proceedings of the 21st Australasian Weeds Conference, Sydney, Australia, 9–13 September 2018.

[56] Ott J.P., Klimešová J., Hartnett D.C. (2019). The ecology and significance of below-ground bud banks in plants. Ann. Bot..

[57] Moretto A.S., Distel R.A. (1998). Requirement of vegetation gaps for seedling establishment of two unpalatable grasses in a native grassland of central Argentina. Austral Ecol..

[58] Hunter H.I. (2014). Declared Plant Policy under the Natural Resources Management Act 2004—Texas Needle Grass (Nassella leucotricha).

[59] Atlas of Living Australia, Weeds Australia Profiles—*Nassella leucotricha* (Trin. & Rupr.) R.W.Pohl. https://profiles.ala.org.au/opus/weeds-australia/profile/Nassella%20leucotricha.

[60] Van Auken O.W. (1997). Germination requirements of aerial chasmogamous florets and seeds of *Nassella leucotricha* (Poaceae). Southwest. Nat..

[61] Pennhall L., James R., Faithfull I. (2000). Landcare Notes—Texas Needlegrass.

[62] Gould F.W. (1978). The grasses of Texas. Plant Prot. Q..

[63] VicFlora, *Nassella leucotricha* (Trin. & Rupr.) Pohl. Royal Botanic Gardens Victoria. https://vicflora.rbg.vic.gov.au/flora/taxon/9064d909-4c6a-4399-b89e-d17d8a378d64.

[64] Leithead H.L., Yarlett L.L., Shiflet T.N. (1971). 100 Native Forage Grasses in 11 Southern States. Agriculture Handbook No. 389.

[65] Webber J., Panetta F.D., Preston C., Watts J.H., Crossman N.D. (2006). Weed risk assessment of the DEH Alert List and other non-native plant species. Managing Weeds in a Changing Climate, Fifteenth Australian Weeds Conference, Adelaide, Australia, 2006.

[66] PIRSA, Have You Seen These Alert Weeds? Government of South Australia. 8 Pages. https://pir.sa.gov.au/__data/assets/pdf_file/0007/347974/54579-19_PIRSA_-_Weed_alert.pdf.

[67] Cook G.D., Dias L. (2006). It was no accident: Deliberate plant introductions by Australian government agencies during the 20th century. Aust. J. Bot..

[68] Atlas of Living Australia, Profiles—*Nassella charruana* (Arechav.) Barkworth. Centre for Invasive Species Solutions, Australian Government. https://profiles.ala.org.au/opus/weeds-australia/profile/Nassella%20charruana.

[69] Stanton R., Burns H.H., Stanton R. (2007). Waking up to sleeper weeds. Agriculture: Opening the Gate. EH Graham Centre Riverina Outlook Conference—Wagga Wagga, Australia, 23 August 2007.

[70] VicFlora, *Nassella charruana* (Arechav.). Royal Botanic Gardens Victoria. https://vicflora.rbg.vic.gov.au/flora/taxon/72180190-f6a5-45f1-94cc-c9b0a693c9e9.

[71] Panario D., Bidegain M. (1997). Climate change effects on grasslands in Uruguay. Clim. Res..

[72] Faithfull I. (1999). Landcare Notes—Lobed Needle Grass.

[73] Vechinno M.C., Blanos V.A., Golluscio R.A., Rodriguez A.M. (2019). Rotational grazing and enclosure improves grassland condition of the halophytic steppe in Flooding Pampa (Argentina) compared with continuous grazing. Rangel. J..

[74] Gardener M.R., Sindel B.M. (1998). The biology of *Nassella* and *Achnatherum* species naturalized in Australia and the implications for management on conservation lands. Plant Prot. Q..

[75] Rosengurtt B., Arrillaga De Maffei B.R., Izaguirre De Artucio P. (1970). Gramineas uruguayas.

[76] Bell M. (2006). Spread of Chilean needle grass (*Nassella neesiana*) in Marlborough New Zealand. N. Z. Plant Prot..

[77] Weeds of Australia, Fact Sheet Index—*Nassalla hyalina*, Biosecurity Queensland Edition, Queensland Government. https://keyserver.lucidcentral.org/weeds/data/media/Html/nassella_hyalina.htm.

[78] Government of South Australia Have You Seen This Alert Weed?—Cane Needlegrass. https://www.pir.sa.gov.au/__data/assets/pdf_file/0020/232445/Nassella_hyalina_June_2011.pdf.

[79] CRC for Australian Weed Management, Commonwealth Department of the Environment and Heritage (2003). Cane Needlegrass—Nassella hyalina, Weed Management Guide.

[80] McLaren D.A., Hunt T.D., Butler K.L. (2016). Effects of spring spot dose response herbicide applications for the control of cane needle grass (*Nassella hyalina*) patches in non-arable situations on the Victorian volcanic plain. Plant Prot. Q..

[81] Atlas of Living Australia, Weeds Australia Profiles—*Nassella hyalina* (Nees) Barkworth. https://profiles.ala.org.au/opus/weeds-australia/profile/Nassella%20hyalina.

[82] Campbell M.H., Barkus B. (1965). The effect of supplementing serrated tussock (*Nassella trichotoma*) with urea and molasses on the live weight of sheep. Aust. J. Exp. Agric..

[83] Badgery W.B., Kemp D., Michalk D.L., King W.M. (2008). Studies of competition between *Nassella trichotoma* (Nees) Hack. ex Arechav. (serrated tussock) and native pastures. 2. Seedling responses. Aust. J. Agric. Res..

[84] Moretto A.S., Distel R.A. (1997). Competitive interactions between palatable and unpalatable grasses native to a temperate semi-arid grassland of Argentina. Plant Ecol..

[85] Gittens C., Ghermandi L., Bran D. (2011). Studying the post-fire performance of tussock grasses in Pantagonia: Survival, biomass production and early competition. J. Arid Environ..

[86] Li R.H., Qiang S. (2007). Progresses and prospects in research of weed seed dispersal. Acta Ecol. Sin..

[87] Victorian Serrated Tussock Working Party Victoria, *Serrated tussock*. http://www.serratedtussock.com/wp-content/uploads/files/WeedHygiene-2020-09-22-web.pdf.

[88] Anderson F., Pettit W., Briese D.T., McLaren D.A. (2002). Biological control of serrated tussock and Chilean needlegrass. Plant Prot. Q..

[89] Hussaini I.P., Lawrie A.C., McLaren D.A. Pathogens on and variation in *Nassella trichotoma* (Poales: Poaceae) in Australia. Proceedings of the X International Symposium on Biological Control of Weeds.

